# Acupuncture to Improve Quality of Life in Patients with Head and Neck Cancer: A Randomized Clinical Trial

**DOI:** 10.3390/cancers18132132

**Published:** 2026-07-01

**Authors:** Agna Soares da Silva Menezes, Gabriela Luize Guimarães Sanches, Cristina Paixão Durães, Larissa Lopes Fonseca, Stephany Gabrielle Chaves Santos, Arlen de Paulo Santiago Filho, Marise Fagundes Silveira, Amanda de Andrade Costa, Gracielle Soares da Silva Ruas, Sérgio Henrique Sousa Santos, Marcos Flávio Silveira Vasconcelos D’Angelo, Alfredo Maurício Batista de Paula, Lucyana Conceição Farias, André Luiz Sena Guimarães

**Affiliations:** 1 Department of Dentistry, Universidade Estadual de Montes Claros (Unimontes), Montes Claros 39401-001, MG, Brazil; 2 Dilson Godinho Hospital, Montes Claros 39400-292, MG, Brazil; 3 Department of Computer Science, Universidade Estadual de Montes Claros (Unimontes), Montes Claros 39401-089, MG, Brazil; 4 Institute of Agricultural Sciences, Universidade Federal de Minas Gerais (UFMG), Montes Claros 39404-547, MG, Brazil

**Keywords:** acupuncture therapy, head and neck neoplasms, quality of life, radiotherapy, squamous cell carcinoma

## Abstract

**Objective:** We aimed to investigate the effects of traditional acupuncture combined with auricular acupressure on quality of life in patients with head and neck squamous cell carcinoma undergoing radiotherapy. **Study Design:** This is a two-arm, parallel, randomized clinical trial with blinded outcome assessment. The study population comprised 107 patients (55 without intervention and 52 with intervention). Data were collected at Dilson Godinho Hospital in Brazil from March 2017 to June 2018, and all patients provided informed consent and were registered under the UTN U1111-1204-8410/RBR-10v5gcdk. Patients in the intervention group received traditional and auricular acupressure in weekly sessions during their radiotherapy treatment, and patients in the control group received no acupuncture. In addition, quality-of-life assessment data were collected using the WHOQOL-BREF instrument. **Results:** Analysis of the scores obtained in the WHOQOL-BREF before and after radiotherapy demonstrated that the use of traditional and auricular acupressure had a positive impact on physical, psychological, social, environmental, and overall quality of life. **Conclusions:** Our findings suggest benefits and provide a context for patients and physicians to decide if acupuncture is a desirable treatment option. However, further studies are required to understand the therapy’s effectiveness better.

## Simple Summary

Head and neck squamous cell carcinoma (HNSCC) and its treatment can significantly impair patients’ quality of life due to physical, psychological, and social burdens associated with radiotherapy. Supportive therapies that reduce treatment-related symptoms and improve well-being are increasingly relevant in cancer care. In this randomized clinical trial, we investigated whether traditional acupuncture combined with auricular acupressure could improve quality of life in patients with HNSCC undergoing radiotherapy. A total of 107 patients were included and evaluated using the WHOQOL-BREF questionnaire before and after treatment. Patients receiving acupuncture demonstrated improvements in physical, psychological, social, environmental, and overall quality-of-life domains, while patients without acupuncture generally showed deterioration across these measures during radiotherapy. No significant adverse events were observed. These findings suggest that acupuncture may represent a safe and supportive complementary intervention for improving quality of life during cancer treatment. However, further studies are needed to confirm long-term benefits and mechanisms of action.

## 1. Introduction

Cancer remains the leading cause of death globally, presenting a formidable barrier to increasing life expectancy across all nations [1]. The global burden of cancer incidence and mortality is escalating rapidly, driven by population aging, growth, and shifts in the prevalence and distribution of significant risk factors. These factors are often linked to socioeconomic development [1].

Head and neck squamous cell carcinoma (HNSCC) represents a critical public health issue [1,2]. Key lifestyle factors contributing to the development and mortality of HNSCC include alcohol consumption and smoking [3,4]. Additionally, evidence suggests that human papillomavirus (HPV) can influence the incidence of this disease [3,5]. While radiotherapy (RT) remains the primary treatment for HNSCC, it is associated with side effects that can significantly impact a patient’s quality of life [6,7]. Patients with HNSCC endure numerous daily challenges, including physical, psychological, spiritual, and emotional difficulties. Comprehensive care often involves palliative therapies to alleviate symptoms and enhance quality of life.

Furthermore, non-pharmacological interventions, such as complementary or integrative therapies including acupuncture, can offer additional benefits to patients undergoing conventional treatments [8,9,10].

Complementary and integrative medicine can address various physical and psychosocial symptoms in cancer patients, such as fatigue, depression, and overall well-being [11]. Nonetheless, well-designed clinical trials are essential to establish the potential benefits of acupuncture [12]. Beyond its potential effects on physical symptoms, acupuncture has been increasingly investigated for its impact on psychological and psychiatric-related outcomes. Previous studies have reported beneficial effects on depression, anxiety, insomnia, psychological distress, and quality of life in different clinical populations [13,14,15], including cancer patients [16]. In addition, neuroimaging studies suggest that acupuncture may modulate brain networks involved in emotional regulation and reward processing, providing a plausible biological basis for its effects on patient-reported well-being [17]. These findings support evaluating quality of life as a clinically relevant outcome when investigating supportive interventions such as acupuncture. The present study investigates the impact of traditional acupuncture combined with auricular acupressure on quality of life in HNSCC patients undergoing radiotherapy.

## 2. Methods

### 2.1. Study Design

The present study is a two-arm, parallel, randomized clinical trial with blinded outcome assessment. The study was reported following the Standards for Reporting Interventions in Clinical Trials of Acupuncture (STRICTA) [12], an adaptation of the Consolidated Standards of Reporting Trials (CONSORT) [18]. Supplementary Materials consists of the CONSORT 2010 Checklist and the STRICTA 2010 Check-list, which are provided to document compliance with the reporting guidelines for randomized clinical trials and acupuncture interventions, respectively.

### 2.2. Ethics and Consent to Participate

All procedures involving human subjects were conducted in accordance with the ethical standards of the institutional and national research committees and the 1964 Declaration of Helsinki and its subsequent amendments, or with comparable ethical standards. Ethical approval for this study was obtained from the Research Ethics Committee of the Universidade Estadual de Montes Claros (UNIMONTES), Brazil, under approval number 1.365.025 (CAAE: 48215415.0.0000.5146), approved on 13 December 2015. A subsequent protocol amendment was also approved by the same Ethics Committee (approval number: 1.736.940; approved on 13 September 2016). The study was registered in the Universal Trial Number system (UTN: U1111-1204-8410) and in the Brazilian Registry of Clinical Trials (ReBEC; registration date: 21 December 2017). All participants provided written informed consent before enrollment in the study.

### 2.3. Sample Size Calculation

**Figure 1:**
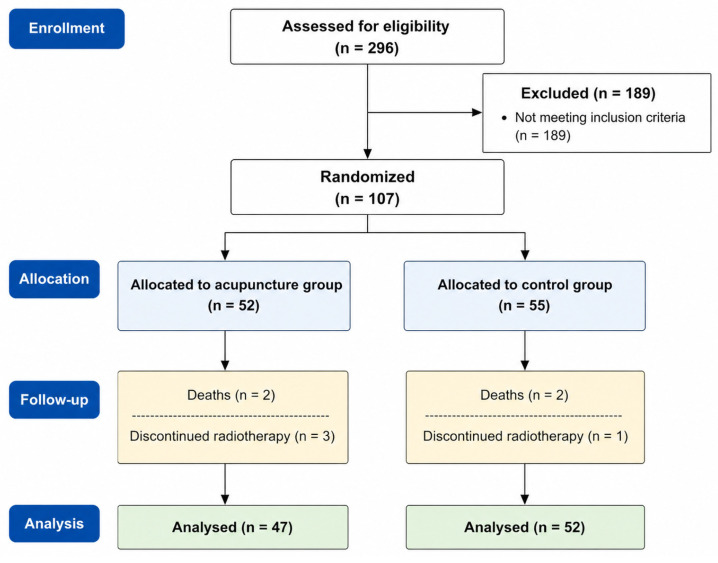
CONSORT flow diagram illustrating participant screening, eligibility assessment, randomization, follow-up, and analysis. A total of 296 patients were assessed for eligibility, of whom 189 were excluded because they did not meet the inclusion criteria. The remaining 107 eligible participants were randomized to the acupuncture group (*n* = 52) or the control group (*n* = 55). During follow-up, two participants in each group died. Additionally, three participants in the acupuncture group and one participant in the control group discontinued radiotherapy before completing the study. Consequently, 47 participants in the acupuncture group and 52 participants in the control group were included in the final per-protocol analysis.

The sample size was computed to achieve a statistical significance level of 0.05 and a statistical power of 0.95, and to estimate overall quality of life. Calculations based on a mean score of 53 and a standard deviation of 4 for the first group and 49 scores for the second group indicate that 94 patients (47 per group) are required. A total of 296 patients were screened for eligibility; 107 (52 with intervention and 55 without intervention) were included in the study (Figure 1). Participant recruitment and data collection were conducted at Dilson Godinho Hospital, Brazil, from March 2017 to June 2018, and all participants provided written informed consent.

### 2.4. Allocation Concealment

Different investigators were responsible for participant recruitment, allocation concealment, intervention delivery, and statistical analysis to minimize the risk of selection bias, in accordance with established recommendations for randomized clinical trials [18,19]. Allocation concealment was performed using a computer-generated randomization table with a 1:1 allocation ratio. Patients and the treating acupuncturist became aware of treatment allocation only after participants had provided written informed consent and had been enrolled in the study. In addition, the investigator responsible for the statistical analyses was not involved in patient recruitment or treatment delivery.

### 2.5. Eligibility Criteria for Inclusion in the Study

Eligible individuals were patients with a histological diagnosis of HNSCC (tongue, gingiva, floor of the mouth, palate, tonsil, oropharynx, pyriform sinus, and hypopharynx), and patients undergoing 3-D RT exclusively or 3-D RT combined with chemotherapy and/or surgery.

### 2.6. Exclusion Criteria

The study excluded patients who had previously undergone radiotherapy for head and neck conditions, patients with SCC lesions associated with ultraviolet light exposure, and patients who declined to participate.

### 2.7. Intervention with Traditional Acupuncture

**Figure 2:**
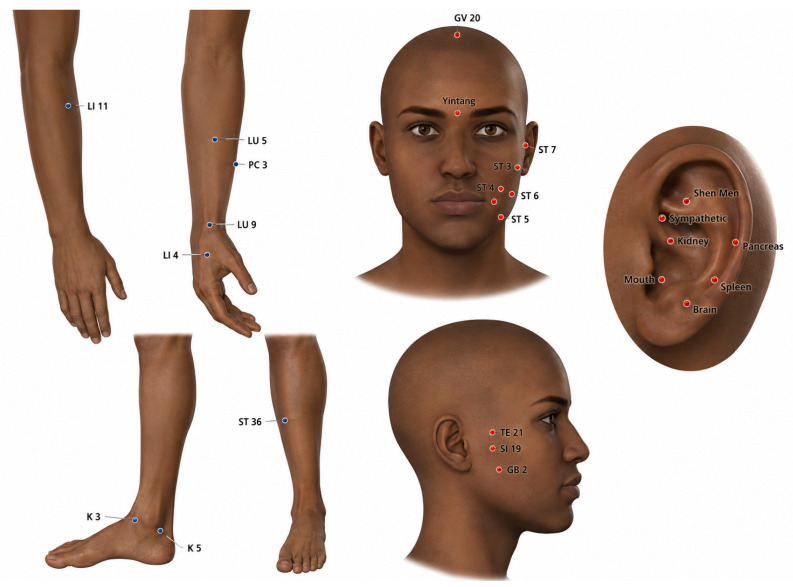
Acupuncture treatment protocol. Representative illustration of the principal body and auricular acupuncture points used in the study. Body points included Yintang (EX-HN3), GV20 (Baihui), ST3 (Juliao), ST4 (Dicang), ST5 (Daying), ST6 (Jiache), ST7 (Xiaguan), GB2 (Tinghui), SI19 (Tinggong), TE21 (Ermen), LI4 (Hegu), LI11 (Quchi), ST36 (Zusanli), LU5 (Chize), LU9 (Taiyuan), PC3 (Quze), K3 (Taixi), and K5 (Shuiquan). Auricular points included Shen Men, Brain, Sympathetic Nervous System, Kidney, Spleen, Pancreas, and Mouth. All patients in the treatment group received the same standardized protocol.

The same acupuncturist performed the treatment on all patients. The acupuncturist is certified in traditional Chinese medicine and has ten years of experience. A list of acupuncture points for the head and limbs was defined [20,21,22]. The local points were Yintang, GV20, ST3, ST4, ST5, ST6, ST7, GB2, SI19, and TE21. The distal points were LI4, LI11, ST36, LU5, LU9, PC3, KI3, and KI5. Auricular acupressure was also performed using the Shenmen, brain, sympathetic, kidney, spleen, pancreas, and mouth points (Figure 2).

All patients in the intervention group received the same treatment: 18 needles applied weekly during radiotherapy. The systemic acupuncture needles (Woo Jeon, Si Group, Gyeonggi-do, Republic of Korea) were sterile, disposable stainless-steel needles (0.25 × 15 mm). Needles were inserted to depths of 5–20 mm, depending on the anatomical location. Needle manipulation was performed to elicit the de qi sensation, which was characterized by a feeling of heaviness, numbness, distension, mild electric-like sensation, or slight discomfort around the needle insertion site. After de qi was achieved, the needles remained in place for 20 min. Manual needle stimulation was repeated every 5 min during the retention period to maintain the de qi response. The needles were removed and discarded after each acupuncture session.

### 2.8. Intervention with Auricular Acupressure

Mustard seeds were used in the auricular points on the left or right ear, and the treated ear was alternated weekly. All seeds were attached to the ears with an antiallergic adhesive for seven days. After seven days, the mustard seeds were removed and discarded. Auricular Acupressure was performed throughout the RT treatment. Patients received weekly instructions on how to stimulate the auricular points.

### 2.9. Patients

The intervention group consisted of patients who received both traditional and auricular acupressure. In comparison, the non-intervention group received no acupuncture.

### 2.10. Instrument

The WHOQOL-BREF was the data-collection instrument used to assess quality of life. WHOQOL-BREF is the abbreviated version of the WHOQOL-100 instrument, which was validated in Portuguese [22]. It consists of an initial part designed to collect socio-demographic and health data, and another on quality of life. This part consists of 26 questions: two on quality of life in general, and the others on each of the facets that compose the original instrument [23]. The questions are organized into the four domains that compose the short version: Physical (pain and discomfort; energy and fatigue; sleep and rest); Psychological (positive feelings; thinking, learning, memory, and concentration; self-esteem; body image and appearance; negative feelings); Social (personal relationships; social support; sexual activity); Environmental (physical safety and security; home environment; financial resources; health and social care: availability and quality; opportunities to acquire new information and skills; participation in, and opportunities for recreation/leisure; physical environment, pollution, traffic noise/climate; transportation). The score for each question ranges from 1 to 5, with higher scores indicating a better assessment [22]. To assess quality of life, the WHOQOL-BREF was administered to participants at the start and end of radiotherapy treatment, in both the acupuncture and non-acupuncture groups.

### 2.11. Outcomes and Measured Parameters

Quality of life was prospectively included in the original study protocol and clinical trial registration and was assessed using the WHOQOL-BREF questionnaire. The present manuscript reports the predefined analysis of these prospectively collected quality-of-life data. The primary outcome of this analysis was the overall quality-of-life score assessed by the WHOQOL-BREF questionnaire. Secondary outcomes included comparisons of the physical, psychological, social, and environmental domains of the WHOQOL-BREF between baseline and post-radiotherapy assessments. Quality-of-life assessments were performed at two predefined time points in both the acupuncture and control groups. The first assessment was conducted before the initiation of radiotherapy to establish baseline comparability between groups, and the second assessment was performed after the completion of radiotherapy to evaluate the impact of the intervention on patient-reported quality of life. The expected trial outcomes and assessment schedule were prospectively defined before participant enrollment and were not modified after trial commencement.

### 2.12. Statistical Analysis

The analyses were conducted per protocol. Participants who died during follow-up or discontinued radiotherapy before completion of the post-treatment WHOQOL-BREF assessment were excluded from the final analyses because complete outcome data were not available. The Kolmogorov–Smirnov and Shapiro–Wilk tests were used to assess the data distribution. The Kolmogorov–Smirnov and Shapiro–Wilk analyses indicated that the data were normally distributed, with values of 0.250 and 0.495 for the asymmetry and kurtosis tests, respectively. Therefore, a repeated-measures ANOVA was performed with one factor of interest: the intervention (acupuncture). The chi-square and Fisher exact tests were used to analyze differences between groups. To facilitate interpretation of the magnitude of the observed effects, partial eta squared (ηp^2^) was calculated as a measure of effect size for repeated-measures ANOVA results. Values of approximately 0.01, 0.06, and 0.14 were interpreted as small, medium, and large effects, respectively. All statistical analyses were performed using the statistical program SPSS (Statistical Package for Social Sciences) v 24.0 for Windows^®^. Results were considered statistically significant at *p* < 0.05.

## 3. Results

Two hundred and ninety-six individuals were initially assessed for eligibility to participate in the study. Of those, 107 met the inclusion criteria and composed the initial sample. Randomization was then performed, with 52 participants allocated to the group receiving traditional and auricular acupressure and 55 to the group not receiving that intervention. During the follow-up, there were four deaths (two in the group that received acupuncture and two in the group without that intervention) and four dropouts from radiotherapy (three in the intervention group and one in the group without that intervention). Therefore, the final sample comprised 47 participants in the Acupuncture group and 52 in the control group (Figure 1). The characterization of the participants (Table 1) showed that the groups were homogeneous with respect to the following variables: treatment performed, chemotherapy regimens, radiotherapy dose, cancer stage and location, gender, age, smoking, and alcoholism.

The non-intervention group comprised 55 patients (43 men and 12 women) aged 37 to 88 years (mean = 61.5; SD = 10.85). Among the patients who did not receive acupuncture, 15 had lesions in the oral cavity, 20 in the oropharynx, and 20 in the hypopharynx. Regarding treatment, 18 patients received only radiotherapy; 14 received a combination of radiotherapy and chemotherapy; 6 received radiotherapy, chemotherapy, and surgery; and 17 received radiotherapy and surgery.

The intervention group comprised 52 patients (47 men and five women) aged 40 to 86 years (mean = 62.7; SD = 11.13). The lesions were distributed as follows: 13 in the oral cavity, 22 in the oropharynx, and 17 in the hypopharynx. Of the total number of patients, 12 were treated only with RT, 11 with RT and chemotherapy, 4 with RT, chemotherapy, and surgery, and 25 with RT and surgery.

No significant differences in quality of life were observed between the groups before treatment (Table 1). However, quality of life assessment performed at the beginning and the end of RT treatment by applying the WHOQOL-BREF instrument showed that the intervention with traditional and auricular acupressure improved overall quality of life (*p* < 0.000) and quality of life in the physical (*p* < 0.000), social (*p* < 0.019), psychological (*p* < 0.002), and environmental (*p* < 0.000) domains.

WHOQOL-BREF mean scores for physical, psychological, social, environmental, and overall quality of life domains were higher in patients who received acupuncture than in the same group before treatment. The group that did not receive acupuncture showed a decline in overall quality of life and in quality of life across all domains following radiotherapy. In the physical domain, the group that received acupuncture showed an increase from 8.25 ± 9.64 before treatment to 8.64 ± 9.76 after RT treatment (*p* < 0.000). The psychological domain was 50.88 ± 10.42 before treatment and 56.47 ± 10.49 after RT treatment with acupuncture (*p* < 0.002). The results for the social domain were 67.55 ± 8.55 before treatment and 71.80 ± 9.60 after RT treatment with acupuncture (*p* < 0.019). The environmental domain showed 53.59 ± 8.39 before treatment and 60.41 ± 7.83 after RT treatment with acupuncture (*p* < 0.000). Finally, overall quality of life was 53.41 ± 6.68 before treatment and 60.76 ± 6.28 after RT with Acupuncture (*p* < 0.000) (Table 2). Effect size analysis indicated that the interaction between the acupuncture intervention and time had large effects on the physical domain (ηp^2^ = 0.262), the environmental domain (ηp^2^ = 0.151), and overall quality of life (ηp^2^ = 0.306). A medium effect was observed for the psychological domain (ηp^2^ = 0.092), and a small-to-medium effect was observed for the social domain (ηp^2^ = 0.056).

## 4. Discussion

In the current study, analysis of scores on the WHOQOL-BREF instrument before and after RT treatment showed that acupuncture use positively affected physical, psychological, environmental, social, and overall quality of life. Acupuncture is a health intervention technology that comprehensively and dynamically addresses the health–disease process in humans and can be used alone or in combination with other therapeutic resources [8,24]. Moreover, acupuncture presents rare adverse effects and sound clinical benefits. It has been widely applied to reduce side effects after RT, but it is not yet recognized as a standard treatment [8,24].

Despite the prevalence and impact on personal and public health, studies assessing therapies to improve the quality of life of HNSCC patients are limited. However, evidence suggests that acupuncture is an alternative with a positive impact on well-being [19,20,21,22] and is also associated with fewer side effects and lower costs of analgesic therapy [25]. Unfortunately, other studies failed to demonstrate significant improvement in emotional, social, and overall quality of life, despite significant improvement in xerostomia symptoms [23,26].

Acupuncture improves symptoms related to cancer and treatment, resulting in a gradual decrease in physical symptoms (fatigue, pain, dysphagia, xerostomia, nausea, numbness, hot flashes), as well as in psychological/emotional symptoms (anxiety, depression, appetite, sleep) [27,28], with an improvement in quality of life after eight weeks of therapy [28,29,30]. Compared to baseline values, the impact on quality of life is maintained 4 weeks after the end of acupuncture sessions, and improvement in patients’ mood is reported at weeks 8 and 12 compared with the assessment before starting therapy [28,29,30]. Although treatment-related symptoms such as xerostomia, dysphagia, mucositis [8], pain, fatigue, and sleep disturbances may substantially affect patient well-being, quality of life represents a broader and more complex construct [31,32,33]. Patient-reported quality of life encompasses not only physical symptoms but also psychological, social, and environmental dimensions [31,32,33]. Consequently, improvements in quality of life cannot be attributed solely to changes in a single symptom [31]. This distinction is particularly relevant in patients with head and neck cancer, whose well-being is influenced by multiple factors, including emotional distress, social interactions, functional limitations, body image, and financial concerns [4,32]. Therefore, multidimensional instruments such as the WHOQOL-BREF provide important complementary information beyond symptom-specific assessments when evaluating supportive interventions in oncology [22].

Pain is one of the most reported physical symptoms in cancer treatment and significantly impacts patients’ quality of life and their tolerance to treatment [6,10]. Studies comparing patients undergoing RT/chemotherapy found that those receiving acupuncture in the head/neck area reduced pain intensity and analgesic use, demonstrating that needle therapy can improve quality of life and reduce side effects [25,29,30]. Acupuncture not only reduces physical symptoms but also impacts social issues. The social domain was the only domain in the present study that did not show statistically significant differences in the effects of acupuncture between the groups at the beginning and the end of RT treatment. However, another study concluded that patients undergoing acupuncture experienced a lower decline in social interactions [34].

A previous study reported that, in addition to improving physical function and reducing the incidence of dyspnea, the use of complementary therapies, such as acupuncture, is associated with less weight loss during treatment, which, in turn, is associated with an improvement in emotional function, since weight loss affects the patient’s self-esteem. In addition, head and neck cancer patients who received complementary and conventional therapies experienced fewer financial problems, reinforcing the positive influence of acupuncture in the environmental domain [34].

Studies have shown clinically significant improvement in well-being after a single acupuncture session, affecting anxiety, depression, xerostomia, fatigue, hot flashes, nausea, numbness/tingling, pain, dyspnea, sleep, and distress. However, among the factors analyzed, appetite showed no statistically significant improvement between baseline and the patient’s first assessment [35].

When comparing patients undergoing sham acupuncture (control group) and real acupuncture (case group), positive changes in quality of life were observed in both groups up to 12 months after the start of treatment, which is clinically meaningful, as it suggests that acupuncture can be effective without requiring continuous use [26]. This outcome can be explained by the fact that simulated acupuncture and tactile stimulation activate various brain areas. In addition, while the action is simulated, some therapists talk to patients for longer and engage in more body contact, thereby psychologically reducing suffering by relieving stress and, in turn, reducing physiological symptoms [24].

A study compared groups that received acupuncture for different periods (2 min, 10 min, and 20 min) and concluded that patients in all three groups perceived improvement in depression, stress, fatigue, and overall quality of life, thus confirming the benefits to well-being regardless of how long the needle is held during the procedure. Therefore, therapy does not depend solely on the time the needle is in place but also on several factors associated with the patient’s clinical condition [33].

Beyond statistical significance, the observed findings may also be clinically relevant. RT for HNSCC is commonly associated with progressive deterioration in quality of life due to treatment-related toxicities and functional impairment. In the present study, patients receiving acupuncture demonstrated improvements in overall quality of life and in the physical, psychological, social, and environmental domains of the WHOQOL-BREF. In contrast, patients in the control group showed declines in overall quality of life and in most domain scores. These contrasting trajectories suggest that acupuncture may help mitigate the expected deterioration in quality of life during radiotherapy and may provide meaningful benefits from the patient’s perspective.

Some limitations should be considered when interpreting the present findings. Although quality of life represents a clinically meaningful patient-reported outcome, the study did not incorporate objective clinical measures such as xerostomia severity, salivary gland function, dysphagia scores, mucositis grading, or nutritional status. In addition, biological and physiological markers were not included; therefore, the mechanisms underlying the observed improvements cannot be established from the present data. Previous studies [13,14,15,16] suggest that acupuncture may influence pain perception, psychological distress, anxiety, depression, sleep quality, and neuroendocrine regulation; however, confirmation of these pathways requires dedicated mechanistic investigations.

Furthermore, complete blinding of participants was not feasible because of the nature of the intervention. Although a sham-acupuncture control was not included, the primary objective of this trial was to evaluate the impact of acupuncture as delivered in a real-world supportive care setting. Consequently, the potential influence of expectancy effects on patient-reported outcomes cannot be completely excluded and should be considered when interpreting the present findings. Finally, quality of life is influenced by physical, psychological, social, and environmental factors. Therefore, the improvements observed in the present study should not be interpreted as resulting from modifications in a single treatment-related symptom. Future studies combining multidimensional quality-of-life assessments with symptom-specific clinical outcomes, biological markers, and appropriate comparator groups may further clarify the mechanisms and broader clinical effects of acupuncture in patients undergoing cancer treatment.

Cancer is a debilitating pathology with treatments that can cause adverse effects on the patient due to their cytotoxic action, impairing and interrupting conventional treatment, and interfering with quality of life. This study showed that traditional and auricular acupressure are effective, safe, efficient, low-cost techniques that expose the patient to minimal risks and positively affect the physical, psychological, environmental, and overall quality of life domains. Only the social domain was not statistically significantly affected in the analyses. The findings suggest benefits and provide a context for patients and physicians to decide if acupuncture is a treatment option. However, further studies are required to understand the therapy’s mechanisms of action, dosage, and effectiveness.

## 5. Conclusions

Traditional and auricular acupressure demonstrated beneficial effects in patients with HNSCC undergoing radiotherapy. The intervention was associated with improvements in physical, psychological, environmental, social, and overall quality-of-life domains. In contrast, patients who did not receive acupuncture generally experienced declines in these outcomes during treatment. Furthermore, no significant adverse events related to the intervention were observed, supporting the safety and tolerability of this complementary approach. These findings suggest that acupuncture may represent a low-risk, potentially valuable supportive strategy to enhance patient well-being during radiotherapy; nevertheless, the absence of a sham-control group and limitations in blinding warrant cautious interpretation. Additional randomized controlled studies with larger sample sizes and longer follow-up periods are needed to further understand the mechanisms of action, long-term effects, and optimal protocols for integrating acupuncture into supportive cancer care.

## Figures and Tables

**Table 1:** The clinical characteristics of the intervention and non-intervention groups.

	No Intervention Group		Intervention Group		
	N 55	%	N52	%	*p*-Value
**Treatment**					
RT	4	57.2%	3	42.8%	
RT + CT	24	57.0%	18	43.0%	
RT + CT + SUR	9	47.40%	10	52.6%	
RT + SUR	18	46.2%	21	53.8%	0.753
**Chemotherapy scheme**					
Cisplatin	31	54.4%	26	45.6%	
Cisplatin and Fluorouracil	1	100.0%	0	0.0%	
Cisplatin and Docetaxel	0	0.0%	1	100.0%	
Taxol	0	0.0%	1	100.0%	
Docetaxel, cisplatin and fluorouracil	1	100.0%	0	0.0%	0.398
**Radiation therapy dose**					
Up to 6000 cGy	3	75.0%	1	25.0%	
Equal or greater than 6000 cGy	52	50.5%	51	49.5%	0.557
Cancer staging					
Stage I	5	55.6%	4	44.4%	
Stage II	1	25.0%	3	75.0%	
Stage III	13	56.5%	10	43.5%	
Stage IV	36	50.7%	35	49.3%	0.698
Anatomical Site					
Oral cavity	15	53.6%	13	46.4%	
Oropharynx	20	47.6%	22	52.4%	
Hypopharynx	20	54.1%	17	45.9%	0.820
Sex					
Male	43	47.8%	47	52.2%	
Female	12	70.6%	5	29.4%	0.084
Age (years)					
Range	37–88		40–86		
Mean (SD)	61.47	(10.85)	62.69	(11.13)	0.567
**Smoking**					
Smoker	52	53.6%	45	46.4%	
Non smoker	3	30.0%	07	70.0%	0.194
Alcoholism					
Alcoholic	49	55.1%	40	44.9%	
Non alcoholic	06	33.3%	12	66.7%	0.122
**General Quality of life**	**Mean**		**Mean**		
	56.4		53.9		0.068

Abbreviations: RT Radiation Therapy. CT Chemotherapy. And SUR Surgery. No differences in clinical features between the intervention and no-intervention groups were observed.

**Table 2:** Comparison of scores obtained in the WHOQOL-BREF of patients undergoing radiotherapy in two groups and relationship with acupuncture intervention. N = 107. Bold values indicate statistically significant differences (*p* < 0.05).

		Group		After Radiotherapy Treatment. Without Intervention with Acupuncture	After Radiotherapy Treatment with Intervention with Acupuncture
		Intervention group	No intervention group	F(gl); valor-*p*	F(gl); valor-*p*
		$\bar{x}$ ± *d.p*	$\bar{x}$ ± *d.p*		
Physicist Domain	Before	49.39 ± 9.64	56.08 ± 8.89	F (1;97) = 1.51 0.223	F(1;97) = 34.45 **<0.000**
	After	57.64 ± 9.76	50.68 ± 8.97		
Psychological Domain	Before	50.88 ± 10.42	53.44 ± 9.14	F(1;97) = 1.25 0.266	F(1;97) = 9.80 **<0.002**
	After	56.47 ± 10.49	50.80 ± 12.35		
Social Domain	Before	67.55 ± 8.55	68.10 ± 10.91	F(1;97) = 1.65 0.202	F(1;97) = 5.73 **<0.019**
	After	71.80 ± 9.60	66.8 ± 10.23		
Environmental Domain	Before	53.59 ± 8.39	55.10 ± 9.01	F(1;97) = 3.49 0.065	F(1;97) = 17.23 **<0.000**
	After	60.41 ± 7.83	52.52 ± 10.16		
General quality of life	Before	53.41 ± 6.68	56.85 ± 6.68	F(1;91) = 3.64	F(1;91) = 40.16
	After	60.76 ± 6.28	52.90 ± 8.63	0.059	**<0.000**

No significant adverse events requiring medical evaluation or specific intervention were reported or observed during the study.

## Data Availability

The datasets generated and/or analyzed during the current study are available from the corresponding author upon reasonable request. Public deposition of the data was not performed due to ethical restrictions and the need to protect participant confidentiality, as required by the approved research protocol.

## Supplementary Materials

The following supporting information can be downloaded at: https://www.mdpi.com/article/10.3390/cancers18132132/s1, CONSORT 2010 Checklist and the STRICTA 2010 Checklist. Reference [18] is cited in Supplementary Materials.

## References

[1] Global Burden of Disease Cancer Collaboration (2019). Global, Regional, and National Cancer Incidence, Mortality, Years of Life Lost, Years Lived with Disability, and Disability-Adjusted Life-Years for 29 Cancer Groups, 1990 to 2017: A Systematic Analysis for the Global Burden of Disease Study. JAMA Oncol..

[2] Santos E.M., Fraga C.A.C., Xavier A., Xavier M.A.S., Souza M.G., Jesus S.F., Paula A.M.B., Farias L.C., Santos S.H.S., Santos T.G. (2021). Prion protein is associated with a worse prognosis of head and neck squamous cell carcinoma. J. Oral Pathol. Med..

[3] Marques-Silva L., Conceição Farias L., de Carvalho Fraga C.A., Macedo de Oliveira M.V., Cardos C.M., Fonseca-Silva T., Cavalieri Gomes C., Batista De-Paula A.M., Gomez R.S., Sena Guimarães A.L. (2012). HPV-16/18 detection does not affect the prognosis of head and neck squamous cell carcinoma in younger and older patients. Oncol. Lett..

[4] Farias L.C., Fraga C.A., De Oliveira M.V., Silva T.F., Marques-Silva L., Moreira P.R., De-Paula A.M., Gomez R.S., Guimaraes A.L. (2010). Effect of age on the association between p16CDKN2A methylation and DNMT3B polymorphism in head and neck carcinoma and patient survival. Int. J. Oncol..

[5] de Paula Souza D.P.S., Dos Reis Pereira Queiroz L., de Souza M.G., de Jesus S.F., Gomes E.S.B., Vitorino R.T., Santos S.H.S., Farias L.C., de Paula A.M.B., D’Angelo M. (2023). Identification of potential biomarkers and survival analysis for oral squamous cell carcinoma: A transcriptomic study. Oral Dis..

[6] Sanches G.L.G., da Silva Menezes A.S., Santos L.I., Duraes C.P., Fonseca L.L., Baldo M.P., de Oliveira Faria T., de Araujo Andrade L.A., Ekel P.I., Santos S.H.S. (2020). Local tissue electrical parameters predict oral mucositis in HNSCC patients: A diagnostic accuracy double-blind, randomized controlled trial. Sci. Rep..

[7] de Souza M.G., de Jesus S.F., Santos E.M., Gomes E.S.B., de Paulo Santiago Filho A., Santos E.M.S., da Silveira L.H., Santos S.H.S., de Paula A.M.B., Farias L.C. (2020). Radiation Therapy Reduced Blood Levels of LDH, HIF-1alpha, and miR-210 in OSCC. Pathol. Oncol. Res..

[8] Menezes A., Sanches G.L.G., Gomes E.S.B., Soares R.G., Duraes C.P., Fonseca L.L., Filho A.P.S., Ribeiro A., Nascimento J.E., Santos S.H.S. (2021). The combination of traditional and auricular acupuncture to prevent xerostomia and anxiety in irradiated patients with HNSCC: A preventive, parallel, single-blind, 2-arm controlled study. Oral Surg. Oral Med. Oral Pathol. Oral Radiol..

[9] Tabosa A.T.L., Souza M.G., de Jesus S.F., Rocha D.F., Queiroz L., Santos E.M., Guimaraes V.H.D., Andrade L.A.A., Santos S.H., de Paula A.M.B. (2022). Effect of low-level light therapy before radiotherapy in oral squamous cell carcinoma: An in vitro study. Pathol. Oncol. Res..

[10] Soares R.G., Farias L.C., da Silva Menezes A.S., de Oliveira E.S.C.S., Tabosa A.T.L., Chagas P.V.F., Santiago L., Santos S.H.S., de Paula A.M.B., Guimaraes A.L.S. (2018). Treatment of mucositis with combined 660- and 808-nm-wavelength low-level laser therapy reduced mucositis grade, pain, and use of analgesics: A parallel, single-blind, two-arm controlled study. Lasers Med. Sci..

[11] Assy Z., Brand H.S. (2018). A systematic review of the effects of acupuncture on xerostomia and hyposalivation. BMC Complement. Altern. Med..

[12] MacPherson H., Altman D.G., Hammerschlag R., Li Y., Wu T., White A., Moher D. (2010). Revised standards for reporting interventions in clinical trials of acupuncture (STRICTA): Extending the CONSORT statement. PLoS Med..

[13] Fan L., Fu W., Chen Z., Xu N., Liu J., Lu A., Su S., Wu T., Ou A. (2016). Curative effect of acupuncture on quality of life in patient with depression: A clinical randomized single-blind placebo-controlled study. J. Tradit. Chin. Med..

[14] Zhao B., Li Z., Wang Y., Ma X., Wang X., Wang X., Liang Y., Yang X., Sun Y., Song M. (2019). Can acupuncture combined with SSRIs improve clinical symptoms and quality of life in patients with depression? Secondary outcomes of a pragmatic randomized controlled trial. Complement. Ther. Med..

[15] Matsuura Y., Hongo S., Taniguchi H., Yasuno F., Sakai T. (2022). Effect of Acupuncture on Physical Symptoms and Quality of Life in Treatment-Resistant Major Depressive Disorder and Bipolar Disorder: A Single-Arm Longitudinal Study. J. Acupunct. Meridian Stud..

[16] Ma F., Zhang H., Li B., Cheng P., Yu M., Wang X. (2023). Acupuncture and moxibustion for malignant tumor patients with psychological symptoms of insomnia, anxiety and depression: A systematic review and Meta-analysis. J. Tradit. Chin. Med..

[17] Wang Z., Wang X., Liu J., Chen J., Liu X., Nie G., Jorgenson K., Sohn K.C., Huang R., Liu M. (2017). Acupuncture treatment modulates the corticostriatal reward circuitry in major depressive disorder. J. Psychiatr. Res..

[18] Schulz K.F., Altman D.G., Moher D., Consort Group (2010). CONSORT 2010 statement: Updated guidelines for reporting parallel group randomised trials. BMJ.

[19] Schulz K.F., Grimes D.A. (2002). Allocation concealment in randomised trials: Defending against deciphering. Lancet.

[20] Blom M., Lundeberg T. (2000). Long-term follow-up of patients treated with acupuncture for xerostomia and the influence of additional treatment. Oral Dis..

[21] Zhuang L., Yang Z., Zeng X., Zhua X., Chen Z., Liu L., Meng Z. (2013). The preventive and therapeutic effect of acupuncture for radiation-induced xerostomia in patients with head and neck cancer: A systematic review. Integr. Cancer Ther..

[22] Fleck M.P., Louzada S., Xavier M., Chachamovich E., Vieira G., Santos L., Pinzon V. (2000). Application of the Portuguese version of the abbreviated instrument of quality life WHOQOL-bref. Rev. Saude Publ..

[23] Wong R.K., Jones G.W., Sagar S.M., Babjak A.F., Whelan T. (2003). A Phase I-II study in the use of acupuncture-like transcutaneous nerve stimulation in the treatment of radiation-induced xerostomia in head-and-neck cancer patients treated with radical radiotherapy. Int. J. Radiat. Oncol. Biol. Phys..

[24] Asadpour R., Meng Z., Kessel K.A., Combs S.E. (2016). Use of acupuncture to alleviate side effects in radiation oncology: Current evidence and future directions. Adv. Radiat. Oncol..

[25] Dymackova R., Selingerova I., Kazda T., Slavik M., Halamkova J., Svajdova M., Slampa P., Slama O. (2021). Effect of Acupuncture in Pain Management of Head and Neck Cancer Radiotherapy: Prospective Randomized Unicentric Study. J. Clin. Med..

[26] Simcock R., Fallowfield L., Monson K., Solis-Trapala I., Parlour L., Langridge C., Jenkins V., ARIX Steering Committee (2013). ARIX: A randomised trial of acupuncture v oral care sessions in patients with chronic xerostomia following treatment of head and neck cancer. Ann. Oncol..

[27] Miller C.S., Johnstone B.M. (2001). Human papillomavirus as a risk factor for oral squamous cell carcinoma: A meta-analysis, 1982–1997. Oral Surg. Oral Med. Oral Pathol. Oral Radiol. Endodontol..

[28] Dean-Clower E., Doherty-Gilman A.M., Keshaviah A., Baker F., Kaw C., Lu W., Manola J., Penson R.T., Matulonis U.A., Rosenthal D.S. (2010). Acupuncture as palliative therapy for physical symptoms and quality of life for advanced cancer patients. Integr. Cancer Ther..

[29] Vinjamury S.P., Li J.T., Hsiao E., Huang C., Hawk C., Miller J., Huang Y. (2013). Effects of acupuncture for cancer pain and quality of life—A case series. Chin. Med..

[30] Mao L., Hong W.K., Papadimitrakopoulou V.A. (2004). Focus on head and neck cancer. Cancer Cell.

[31] Bhardwaj T. (2021). Quality of Life of Head and Neck Cancer Patients: Psychosocial Perspective using Mixed Method Approach. Indian J. Palliat. Care.

[32] Cerea S., Sansoni M., Scarzello G., Groff E., Ghisi M. (2022). Psychological variables associated with quality of life in patients with head and neck cancer: The role of body image distress. Support Care Cancer.

[33] Oh B., Eade T., Kneebone A., Pavlakis N., Clarke S., Eslick G., River J., Back M. (2017). Factors affecting whether or not cancer patients consider using acupuncture. Acupunct. Med..

[34] Hsieh P.C., Yang M.C., Wu Y.K., Chen H.Y., Tzeng I.S., Hsu P.S., Lee C.T., Chen C.L., Lan C.C. (2019). Acupuncture therapy improves health-related quality of life in patients with chronic obstructive pulmonary disease: A systematic review and meta-analysis. Complement. Ther. Clin. Pract..

[35] Lopez G., Garcia M.K., Liu W., Spano M., Underwood S., Dibaj S.S., Li Y., Moguel R., Williams J., Bruera E. (2018). Outpatient acupuncture effects on patient self-reported symptoms in oncology care: A retrospective analysis. J. Cancer.

