# Supplementary material for: Acupuncture to Improve Quality of Life in Patients with Head and Neck Cancer: A Randomized Clinical Trial
Source: Cancers (Basel). 2026 Jul 1;18(13):2132. doi: 10.3390/cancers18132132 (PMC13359815; doi:10.3390/cancers18132132)
Supplement: Supplementary file 1 [file cancers-18-02132-s001.zip › supplementary material/CONSORT-2010-Checklist manuscript.pdf]

# CONSORT 2010 checklist of information to include when reporting a randomised trial*

| Section/Topic | Item No | Checklist Item | Page(s) | Line(s) |
|---|---|---|---|---|
| Title and Abstract | 1a | Identification as a randomised trial in the title | 1 | 1–2 |
| | 1b | Structured summary of trial design, methods, results, and conclusions | 1–2 | 16–30 |
| Introduction | 2a | Scientific background and rationale | 2–4 | 33–78 |
| | 2b | Specific objectives or hypotheses | 4 | 74–78 |
| Methods | 3a | Description of trial design including allocation ratio | 5 | 90–94 |
| | 3b | Important changes to methods after trial commencement | NA | NA |
| Participants | 4a | Eligibility criteria for participants | 6 | 117–123 |
| | 4b | Settings and locations where the data were collected | 4–5 | 80–116 |
| Interventions | 5 | Interventions for each group with sufficient details to allow replication | 6–10 | 124–224 |
| Outcomes | 6a | Completely defined pre-specified primary and secondary outcome measures | 10–15 | 225–347 |
| | 6b | Any changes to trial outcomes after the trial commenced | NA | NA |
| Sample size | 7a | How sample size was determined | 5 | 95–103 |
| | 7b | Interim analyses and stopping guidelines | NA | NA |
| Randomisation: Sequence generation | 8a | Method used to generate the random allocation sequence | 5 | 104–107 |
| | 8b | Type of randomisation; details of any restriction | 5 | 104–107 |
| Allocation concealment mechanism | 9 | Mechanism used to implement the random allocation sequence | 5 | 104–110 |
| Implementation | 10 | Who generated the allocation sequence, enrolled participants, and assigned participants | 5 | 104–110 |
| Blinding | 11a | Who was blinded after assignment to interventions and how | 5 | 106–107 |
| | 11b | Description of similarity of interventions | 6–7 | 124–158 |
| Statistical methods | 12a | Statistical methods used to compare groups | 15 | 348–356 |
| | 12b | Methods for additional analyses | 15 | 348–356 |
| Results | 13a | Participant flow (numbers randomly assigned, treated, analysed) | 21–22 | 493–515 |
| | 13b | Losses and exclusions after randomisation | 21–22 | 493–515 |
| Recruitment | 14a | Dates defining recruitment and follow-up periods | 4, 10–11 | 80–89; 226– |

| Section/Topic | Item No | Checklist Item | Page(s) | Line(s) |
|---|---|---|---|---|
| | | | | 244 |
| | 14b | Why the trial ended or was stopped | NA | NA |
| Baseline data | 15 | Baseline demographic and clinical characteristics | 21–22 | 493–515 |
| Numbers analysed | 16 | Number of participants included in each analysis | 21–30 | 493–697 |
| Outcomes and estimation | 17a | Results for each primary and secondary outcome | 21–30 | 493–697 |
| | 17b | Binary outcomes and effect sizes | 24–25 | 561–587 |
| Ancillary analyses | 18 | Results of any other analyses performed | 24–30 | 561–697 |
| Harms | 19 | Important harms or unintended effects | 24–25 | 561–587 |
| Discussion | 20 | Trial limitations | 17–18 | 401–430 |
| | 21 | Generalisability of the trial findings | 17–19 | 401–457 |
| | 22 | Interpretation consistent with results | 16–19 | 376–457 |
| Other Information | 23 | Registration number and name of trial registry | 4 | 80–84 |
| | 24 | Where the full trial protocol can be accessed | NA | NA |
| | 25 | Sources of funding and role of funders | 20 | 484–488 |

Citation: Schulz KF, Altman DG, Moher D, for the CONSORT Group. CONSORT 2010 Statement: updated guidelines for reporting parallel group randomised trials. BMC Medicine. 2010;8:18.
© 2010 Schulz et al. This is an Open Access article distributed under the terms of the Creative Commons Attribution License (http://creativecommons.org/licenses/by/2.0), which permits unrestricted use, distribution, and reproduction in any medium, provided the original work is properly cited.

*We strongly recommend reading this statement in conjunction with the CONSORT 2010 Explanation and Elaboration for important clarifications on all the items. If relevant, we also recommend reading CONSORT extensions for cluster randomised trials, non-inferiority and equivalence trials, non-pharmacological treatments, herbal interventions, and pragmatic trials. Additional extensions are forthcoming: for those and for up-to-date references relevant to this checklist, see www.consort-statement.org.
