# Supplementary material for: Acupuncture to Improve Quality of Life in Patients with Head and Neck Cancer: A Randomized Clinical Trial
Source: Cancers (Basel). 2026 Jul 1;18(13):2132. doi: 10.3390/cancers18132132 (PMC13359815; doi:10.3390/cancers18132132)
Supplement: Supplementary file 1 [file cancers-18-02132-s001.zip › supplementary material/STRICTA-2010-Checklist manuscript.pdf]

**STandards for Reporting Interventions in Clinical Trials of Acupuncture (STRICTA): extending the CONSORT Statement**

**Table 1: STRICTA 2010 checklist of information to include when reporting interventions in a clinical trial of acupuncture (Expansion of Item 5 from CONSORT 2010 checklist)**

| Item | Detail | Page(s) | Line(s) |
|---|---|---|---|
| 1. Acupuncture rationale | 1a) Style of acupuncture (Traditional Chinese Medicine, auricular acupuncture) | 6–7 | 123–158 |
| | 1b) Reasoning for treatment provided, based on historical context, literature sources, and/or consensus methods | 3–4 | 58–78 |
| | 1c) Extent to which treatment was varied | 7–8 | 146–171 |
| 2. Details of needling | 2a) Number of needle insertions per subject per session | 7 | 133–136 |
| | 2b) Names (or location if no standard name) of points used (uni/bilateral) | 6–7 | 127–135 |
| | 2c) Depth of insertion | 7 | 137–139 |
| | 2d) Response sought (e.g. de qi or muscle twitch response) | 7 | 139–142 |
| | 2e) Needle stimulation (e.g. manual, electrical) | 7 | 143–145 |
| | 2f) Needle retention time | 7 | 142–143 |
| | 2g) Needle type (diameter, length, manufacturer, or material) | 7 | 136–138 |
| 3. Treatment regimen | 3a) Number of treatment sessions | 7–8 | 133–171 |
| | 3b) Frequency and duration of treatment sessions | 7–8 | 133–171 |
| 4. Other components of treatment | 4a) Details of other interventions administered to the acupuncture group | 8 | 159–171 |
| | 4b) Setting and context of treatment, including instructions to practitioners and patients | 8 | 159–171 |
| 5. Practitioner background | 5) Description of participating acupuncturists (qualification, years in practice, experience) | 6 | 124–126 |
| 6. Control or comparator interventions | 6a) Rationale for the control or comparator in the context of the research question | 8–9 | 172–176 |
| | 6b) Precise description of the control or comparator | 8–9 | 172–176 |

Observation: The manuscript adequately reports the acupuncture intervention according to the STRICTA recommendations, including acupuncture rationale, needling details, treatment regimen, practitioner background, and comparator description.
